# Intergenerational transmission of maternal behavioral traits in mice: involvement of the gut microbiota

**DOI:** 10.3389/fnins.2023.1302841

**Published:** 2024-01-08

**Authors:** Kazutaka Mogi, Uruma Akiyama, Natsumi Futagawa, Kanami Tamura, Mai Kamiya, Mami Mizuta, Miku Yamaoka, Itsuka Kamimura, Sayaka Kuze-Arata, Takefumi Kikusui

**Affiliations:** Department of Animal Science and Biotechnology, Azabu University, Sagamihara, Japan

**Keywords:** maternal behavioral traits, licking/grooming, intergenerational transmission, gut microbiota, germ-free mice

## Abstract

The matrilineal transmission of maternal behavior has been reported in several species. Studies, primarily on rats, have suggested the importance of postnatal experience and the involvement of epigenetic mechanisms in mediating these transmissions. This study aims to determine whether the matrilineal transmission of maternal behavior occurs in mice and whether the microbiota is involved. We first observed that early weaned (EW) female mice showed lower levels of maternal behavior, particularly licking/grooming (LG) of their own pups, than normally weaned (NW) female mice. This difference in maternal behavioral traits was also observed in the second generation, even though all mice were weaned normally. In the subsequent cross-fostering experiment, the levels of LG were influenced by the nurturing mother but not the biological mother. Finally, we transplanted the fecal microbiota from EW or NW mice into germ-free (GF) mice raising pups. The maternal behaviors that the pups exhibited toward their own offspring after growth were analyzed, and the levels of LG in GF mice colonized with microbiota from EW mice were lower than those in GF mice colonized with microbiota from NW mice. These results clearly indicate that, among maternal behavioral traits, LG is intergenerationally transmitted in mice and suggest that the vertical transmission of microbiota is involved in this process. This study demonstrates the universality of the intergenerational transmission of maternal behavioral traits and provides new insights into the role of microbiota.

## 1 Introduction

Matrilineal transmission of maternal behavior occurs in several mammalian species. In humans, an intergenerational transmission of maternal care and overprotection, measured using the Parental Bonding instrument, was shown to occur between women and their daughters (Miller et al., 1997). This transmission is independent of the socioeconomic status and maternal or daughter temperament. A human longitudinal study on the transmission of attachment across three generations reported that mothers’ attachment classifications were a good predictor of their infants’ or grandmothers’ attachment classifications (Benoit and Parker, 1994). Primates have similarities with humans. Longitudinal observations of vervet monkeys living in naturally composed captive social groups showed that the frequency of mother–infant contact in the first 6 months of life could be predicted based the amount of mother–infant contact females had experienced as infants (Fairbanks, 1989). It was also demonstrated that rhesus monkeys exposed to abusive parenting had a high probability of engaging in abusive parenting in adulthood (Maestripieri and Carroll, 1998). Furthermore, cross-fostering offspring experiments suggested that the intergenerational transmission of infant abuse in rhesus monkeys was the result of early postnatal experience and not genetic inheritance (Maestripieri, 2005).

Matrilineal transmission of maternal behavior has also been reported in rodents, which are fecund and can be used to study the behavior of multiple generations of offspring in a short period of time. In particular, studies on Long-Evans rats have provided substantial insights. The frequency with which dams engage in pup licking/grooming (LG) during the first week postpartum varies considerably among individuals; however, it shows a high level of stability within individuals and can be characterized as engaging in high or low levels of maternal LG (Champagne et al., 2003). The offspring of high- and low-LG dams exhibit LG levels that are highly correlated with the behavior of their mother (Francis et al., 1999; Champagne et al., 2003). Furthermore, cross-fostering experiments in pups have demonstrated that LG frequency when offspring are mothers is more similar to that of the nurturing mother than that of the biological mother (Francis et al., 1999; Champagne et al., 2003), thereby suggesting the importance of early postnatal experience. It has also been suggested that epigenetic mechanisms mediate the transmission of maternal behavioral traits. High levels of maternal LG received in infant period are associated with decreased ERα promoter methylation in the medial preoptic area (MPOA), which regulates maternal behavior (Champagne et al., 2006). This subsequently increases the levels of oxytocin receptor binding in the MPOA in adulthood, which increases the LG frequency (Francis et al., 2000; Champagne et al., 2001).

This study firstly aimed to determine whether the matrilineal transmission of maternal behavior occurs in mice. There have been some reports examining the matrilineal transmission of maternal behavior in mice, but it is not yet clear whether this exists (Rymer and Pillay, 2011; Rymer and Pillay, 2013; Sauce et al., 2017). We have previously demonstrated that mouse pups can distinguish between their own mother and an alien mother and that mothers can also differentiate their own pups from alien pups (Mogi et al., 2017), thereby suggesting the existence of mother–infant bonding. Furthermore, as a result of the deprivation of mother–infant bonding owing to early weaning, early weaned (EW) F1 mother mice showed a lower frequency of LG in their own pups than the normally weaned (NW) F1 mother mice (Kikusui et al., 2005; Sakamoto et al., 2021). In this study, we compared maternal behavior between F2 offspring of EW F1 mothers (EW-F2 offspring) and F2 offspring of NW F1 mothers (NW-F2 offspring). We also performed cross-fostering experiments on the F2 offspring to test the importance of the postnatal environment.

Furthermore, we focused on the involvement of the microbiota in the intergenerational transmission of maternal behavioral traits in this study. The microbiota is an important regulator of nervous system development and function and several host behaviors (Vuong et al., 2017). Animals reared without microbial colonization (germ-free, GF) exhibit altered social behavior and neurophysiology compared to those of conventionally colonized (specific pathogen-free, SPF) controls (Desbonnet et al., 2014; Kamimura et al., 2019; Wu et al., 2021). Studies on maternal challenges, such as immune activation, a high-fat diet, or psychological stress (Buffington et al., 2016; Kim et al., 2017; Jašarević et al., 2018; Di Gesù et al., 2022), have suggested that the altered maternal gut microbiome is involved in the regulation of the developmental processes of offspring that affect brain function and behavior in adulthood. Notably, maternal dysbiosis of the gut microbiota caused by high-fat diet is vertically transmitted to offspring, and this induces social deficit in adulthood (Buffington et al., 2016; Di Gesù et al., 2022). To test our hypothesis that maternal microbiota could mediate the intergenerational transmission of maternal behavioral traits, we transplanted fecal microbiota from NW or EW mice into GF mice raising pups. After the pups had grown, their maternal behavior was observed, and differences in maternal behavioral traits were compared between GF mice colonized with the NW and EW microbiota.

## 2 Materials and methods

### 2.1 Animals

C57BL/6J mice were used in experiments 1 and 2; GF mice were used in experiment 3. All mice were maintained under a standard 12 h:12 h light–dark cycle (lights on at 6 am) and provided a pelleted diet and water *ad libitum*. The environment was maintained at a constant temperature (24 ± 1°C) and humidity (50 ± 5%). The animal studies were reviewed and approved by Committee of Azabu University (#180316–7, #200312–23, #210319–30).

C57BL/6J mice were obtained from CLEA Japan Inc. (Tokyo, Japan) and bred in our laboratory. For breeding experiments, adult male and female mice were pair-housed, and male mice were separated from females as soon as the vaginal plug was confirmed. Therefore, only mother mice reared litters in the experiments. Each litter was culled to 6–8 pups with a balanced number of males and females.

Pregnant GF mice were obtained from CLEA Japan and housed in vinyl isolators (Sanki Kagaku Kougei Co., Kanagawa, Japan). All GF mice were fed sterile pellet diet (CMF 50 kGy, ORIENTAL YEAST, Tokyo, Japan) and sterile water. Immediately after transferring pregnant GF mice to a vinyl isolator, swab tests were performed as previously described (Kamimura et al., 2019) to ensure a GF state. The vinyl isolator was swabbed with an ICR-swab (Merck Millipore, Darmstadt, Alemanha) and then, incubated at 25°C for 48 h, and turbidity was measured using an absorptiometer (Shimazu CO., Kyoto, Japan). Each GF litter was culled to 6–8 pups with a balanced number of males and females and used in Experiment 3.

### 2.2 Experiment 1: observations of maternal behavioral traits across two generations

**Figure 1:**
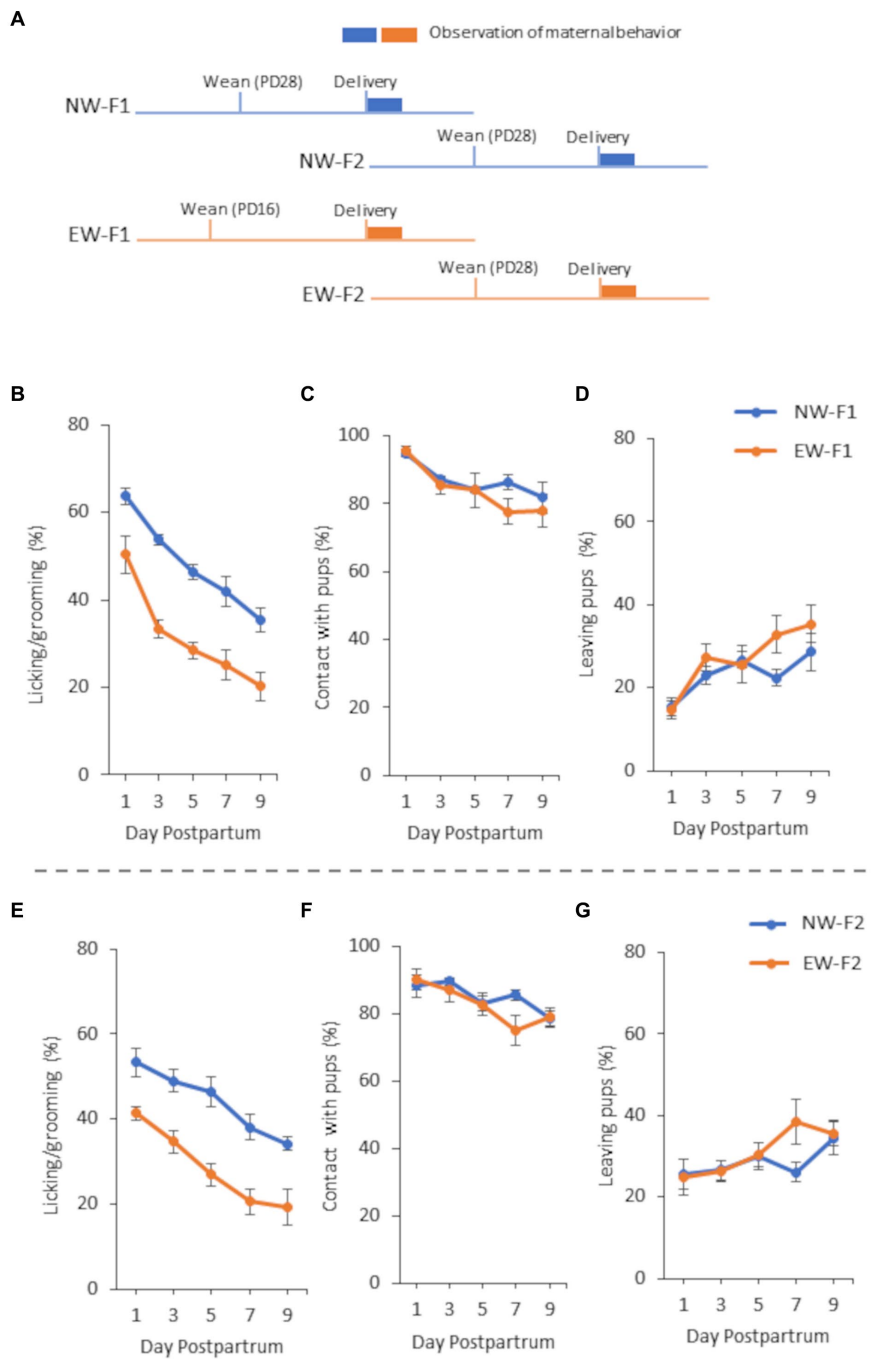
A schematic diagram of the experimental schedule **(A)** and maternal behavior of F1 females **(B–D)** and F2 females **(E–G)** from 1 to 9 days postpartum in Experiment 1. **(A)** NW-F1 and EW-F1 mice were weaned on PD28 and PD16, respectively, whereas both NW-F2 and EW-F2 mice were weaned on PD28. **(B)** Incidence of “licking/grooming” in NW-F1 and EW-F1 mice. Group [*F*(1, 12.19) = 35.24, *p* < 0.0001], Day [*F*(4, 46.4) = 60.35, *p* < 0.0001], and Group × Day interaction [*F*(4, 46.4) = 0.93, *p* = 0.45]. **(C)** Incidence of “contact with pups” in NW-F1 and EW-F1 mice. Group [*F*(1, 12.32) = 1.39, *p* = 0.26], Day [*F*(4, 46.87) = 7.86, *p* < 0.0001], and Group × Day interaction [*F*(4, 46.87) = 0.72, *p* = 0.58]. **(D)** Incidence of “leaving pups” in NW-F1 and EW-F1 mice. Group [*F*(1, 12.23) = 2.75, *p* = 0.12], Day [*F*(4, 47.53) = 7.26, *p* < 0.0001], and Group × Day interaction [*F*(4, 47.53) = 1.08, *p* = 0.38]. **(E)** Incidence of “licking/grooming” in NW-F2 and EW-F2 mice. Group [*F*(1, 8) = 20.20, *p* = 0.0020], Day [*F*(4, 32) = 42.04, *p* < 0.0001], and Group × Day interaction [*F*(4, 32) = 1.22, *p* = 0.32]. **(F)** Incidence of “contact with pups” in NW-F2 and EW-F2 mice. Group [*F*(1, 8) = 0.55, *p* = 0.48], Day [*F*(4, 32) = 8.31, *p* = 0.0001], and Group × Day interaction [*F*(4, 32) = 2.28, *p* = 0.08]. **(G)** Incidence of “leaving pups” in NW-F2 and EW-F2 mice. Group [*F*(1, 8) = 0.67, *p* = 0.44], Day [*F*(4, 32) = 3.34, *p* = 0.022], and Group × Day interaction [*F*(4, 32) = 1.59, *p* = 0.20].

The procedure used to obtain EW mice (F1) was the same as that described in our previous studies (Mogi et al., 2016; Sakamoto et al., 2021). Pregnant female mice were monitored daily until parturition. For each litter, the date of birth was designated postnatal day 0 (PD0). On PD16, half of the litter was separated from each dam and assigned to the EW group. The remaining pups were assigned to the NW group, cared for using standard procedures, and weaned on PD28 (Figure 1A). EW mice were fed powdered pellets until PD28. After weaning, they were fed regular pellets, similar to that used for the NW mice. After weaning, two or three pups were placed together in cages according to their original group and sex. When both EW and NW F1 female mice [EW-F1 mice (*n* = 7) and NW-F1 mice (*n* = 7)] were 8 weeks old, each female was mated with an NW male mouse. Maternal behavior of female mice after birth was evaluated (see section 2.5). All F2 litters were NW on PD28 and bred, as described above (Figure 1A). When F2 females from EW and NW F1 dams [EW-F2 mice (*n* = 5) and NW-F2 mice (*n* = 5), respectively] were 8 weeks old, each female was mated with an NW male mouse, and their maternal behavior was observed using the method used for F1 female mice.

### 2.3 Experiment 2: cross-fostering experiments on F2 female offspring

**Figure 2:**
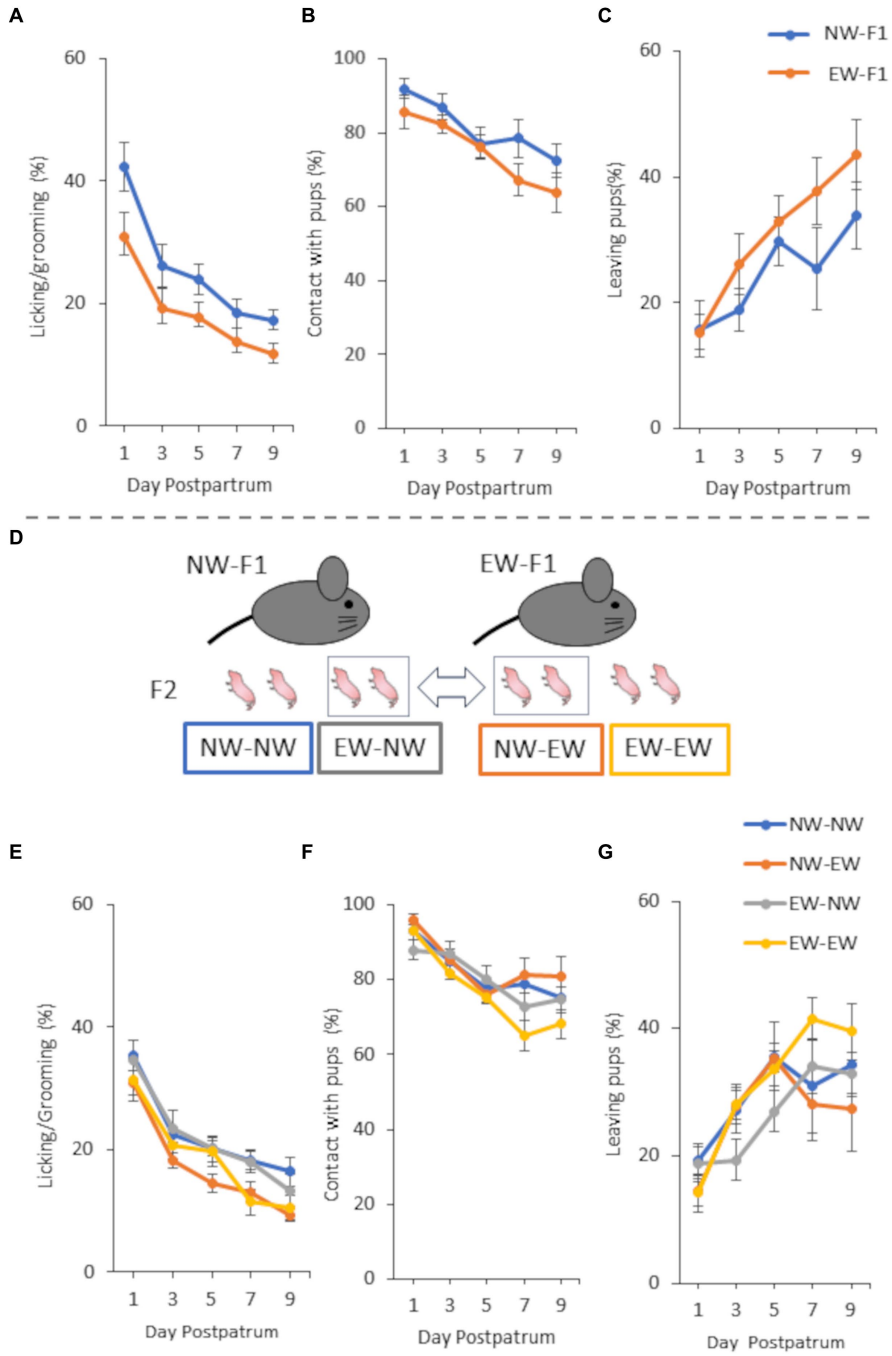
Maternal behavior of F1 females **(A–C)** and F2 females **(E–G)** from 1 to 9 days postpartum and a schematic diagram of cross-fostering **(D)** in Experiment 2. **(A)** Incidence of “licking/grooming” in NW-F1 and EW-F1 mice. Group [*F*(1, 10) = 5.84, *p* = 0.036], Day [*F*(4,40) = 52.63, *p* < 0.0001], and Group × Day interaction [*F*(4, 40) = 0.21, *p* = 0.93]. **(B)** Incidence of “contact with pups” in NW-F1 and EW-F1 mice. Group [*F*(1, 11) = 1.47, *p* = 0.25], Day [*F*(4, 44) = 13.99, *p* < 0.0001], and Group × Day interaction [*F*(4, 44) = 0.66, *p* = 0.62]. **(C)** Incidence of “leaving pups” in NW-F1 and EW-F1 mice. Group [*F*(1, 11) = 1.68, *p* = 0.22], Day [*F*(4, 44) = 13.09, *p* < 0.0001], and Group × Day interaction [*F*(4, 44) = 0.92, *p* = 0.46]. **(D)** A part of the female litter was reciprocally cross-fostered to mothers of other weaned groups within 24 h after birth. **(E)** Incidence of “licking/grooming” in cross-fostering experiment. Prenatal mother [*F*(1, 20) = 0.0061, *p* = 0.94], Postnatal mother [*F*(1, 20) = 6.65, *p* = 0.018], Day [*F*(4, 80) = 81.38, *p* < 0.0001], Prenatal mother × Postnatal mother interaction [*F*(1, 20) = 0.34, *p* = 0.57], Prenatal mother × Day interaction [*F*(4, 80) = 1.25, *p* = 0.30], Postnatal mother × Day interaction [*F*(4, 80) = 2.42, *p* = 0.055], and Prenatal mother × Postnatal mother × Day interaction [*F*(4, 80) = 1.17, *p* = 0.33]. **(F)** Incidence of “contact with pups” in cross-fostering experiment. Prenatal mother [*F*(1, 20) = 1.72, *p* = 0.20], Postnatal mother [*F*(1, 20) = 0.086, *p* = 0.77], Day [*F*(4, 80) = 29.31, *p* < 0.0001], Prenatal mother × Postnatal mother interaction [*F*(1, 20) = 0.71, *p* = 0.41], Prenatal mother × Day interaction [*F*(4, 80) = 2.77, *p* = 0.033], Postnatal mother × Day interaction [*F*(4, 80) = 1.082, *p* = 0.37], and Prenatal mother × Postnatal mother × Day interaction [*F*(4, 80) = 0.94, *p* = 0.44]. **(G)** Incidence of “leaving pups” in cross-fostering experiment. Prenatal mother [*F*(1, 20) = 0.075, *p* = 0.79], Postnatal mother [*F*(1, 20) = 0.12, *p* = 0.73], Day [*F*(4, 80) = 17.48, *p* < 0.0001], Prenatal mother × Postnatal mother interaction [*F*(1, 20) = 1.54, *p* = 0.23], Prenatal mother × Day interaction [*F*(4, 80) = 2.65, *p* = 0.039], Postnatal mother × Day interaction [*F*(4, 80) = 1.092, *p* = 0.37], and Prenatal mother × Postnatal mother × Day interaction [*F*(4, 80) = 0.49, *p* = 0.75].

A group of mice different from that used in Experiment 1 was used for Experiment 2. Similar to Experiment 1, both EW-F1 (*n* = 6) and NW-F1 mice (*n* = 6) were generated, and each female was paired with an NW male mouse. Subsequently, cross-foster operations were performed with female offspring. In previous intrastrain cross-fostering studies, in which all of littermates were cross-fostered within C57BL/6J mice, one study reported that foster dams showed more frequent LG to pups as compared with biological dams (van der Veen et al., 2008), but another study reported that foster dams showed lesser LG to pups and left from pups more frequently (Cox et al., 2013). To minimize the potential influence of cross-fostering effect, the present study was conducted without cross-fostering all of littermate as follows. When EW-F1 and NW-F1 mice gave birth on the same day (within 24 h), some of the female litter was reciprocally cross-fostered with mothers from other weaned groups (Figure 2D). F2 female offspring with different real and foster mothers were generated [NW-EW mice (*n* = 7) and EW-NW mice (*n* = 5): the naming convention for groups in cross-fostering experiments is that the first name refers to the prenatal mother and the second name refers to the postnatal mother]. Control mice were handled in the same manner as that of the pups, but were returned to their own parents [NW-NW mice (*n* = 7) and EW-EW mice (*n* = 5]). The maternal behavior of F1 female mice was determined using the method used for Experiment 1. Each F2 female mouse was mated with an NW male mouse, and maternal behavior was observed using the method used for Experiment 1.

### 2.4 Experiment 3: transplantation of fecal microbiota into GF mice

**Figure 3:**
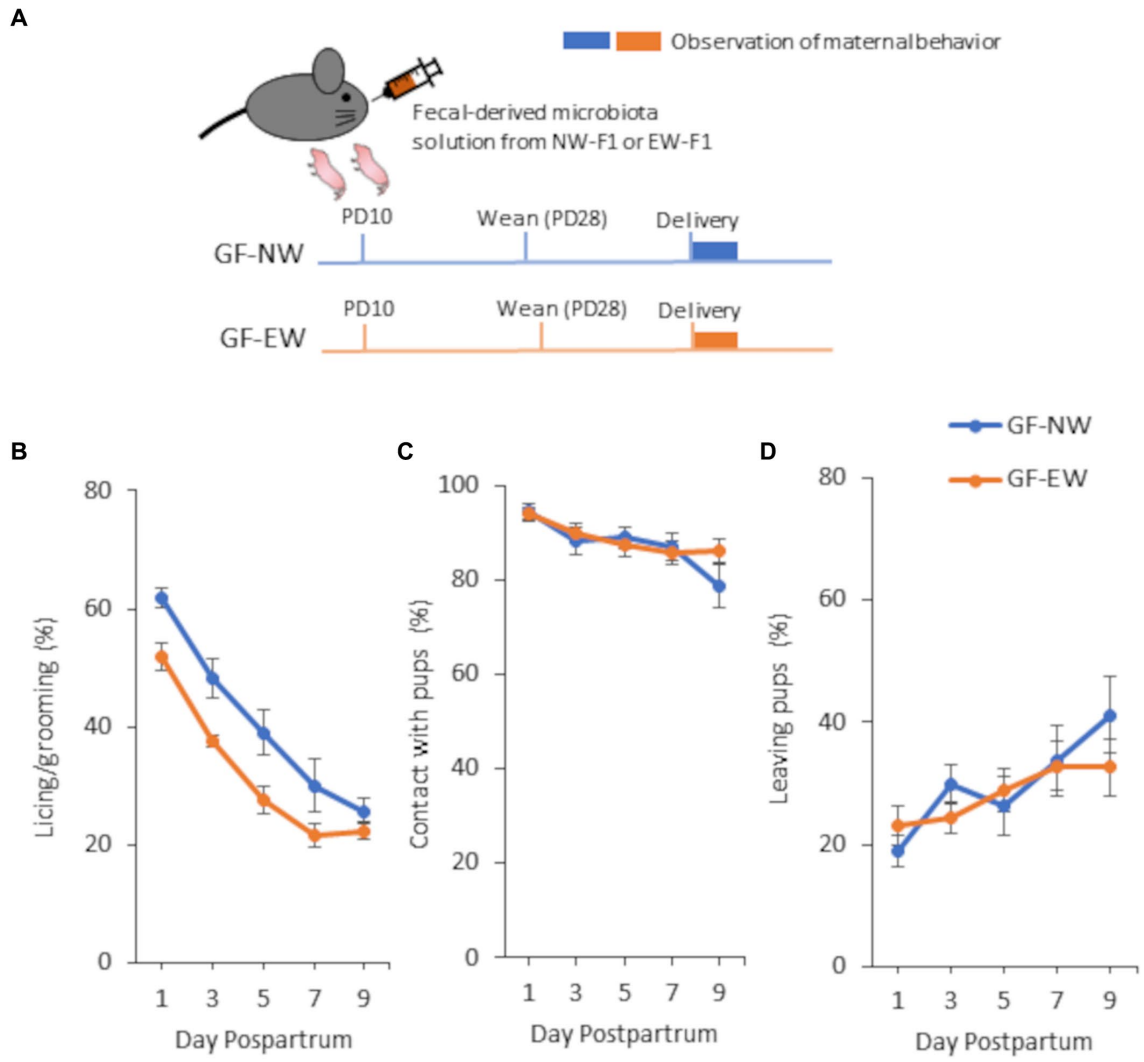
A schematic diagram of the experimental schedule **(A)** and maternal behavior of GF-NW and GF-EW females **(B–D)** from 1 to 9 days postpartum in Experiment 3. **(A)** For colonization of GF pups with microbiota, fecal-derived microbiota solution of EW-F1 or NW-F1 mice was orally administrated to GF dam. **(B)** Incidence of “licking/grooming” in GF-NW and GF-EW mice. Group [*F*(1, 19.83) = 10.86, *p* = 0.0036], Day [*F*(4, 77.3) = 42.39, *p* < 0.0001], and Group × Day interaction [*F*(4, 77.3) = 0.49, *p* = 0.74]. **(C)** Incidence of “contact with pups” in GF-NW and GF-EW mice. Group [*F*(1, 20.27) = 0.091, *p* = 0.77], Day [*F*(4, 77.55) = 7.63, *p* < 0.0001], and Group × Day interaction [*F*(4, 77.55) = 1.32, *p* = 0.27]. **(D)** Incidence of “leaving pups” in GF-NW and GF-EW mice. Group [*F*(1, 20.18) = 0.15, *p* = 0.70], Day [*F*(4, 77.58) = 4.41, *p* = 0.0029], and Group × Day interaction [*F*(4, 77.58) = 0.93, *p* = 0.45].

When the GF pups were on PD10, each GF dam was orally administered with a fecal-derived microbiota solution (see section 2.6) from EW-F1 or NW-F1 mice [GF-EW mice (*n* = 14) and GF-NW mice (*n* = 8), respectively]. This process establishes bacterial flora in the mothers of each cage, which in turn establishes the same flora in the pups (Figure 3A). After weaning on PD28, two or three pups were placed together in cages according to their original group and sex in a vinyl isolator. When both GF-EW and GF-NW female mice were 8 weeks old, each female was mated with a male mouse of the same group from a different dam. One week after mating, the female mice were separated from the male mice. The maternal behavior of the female mice was evaluated after giving birth, using the same method used for Experiments 1 and 2.

### 2.5 Maternal behavior observations

This procedure was similar to that used in our previous study (Sakamoto et al., 2021). Maternal behaviors were digitally videotaped on postpartum days 1, 3, 5, 7, and 9 (Supplementary Video S1). Six observations were performed per day of 60 min each during the light cycle (6–7 am, 8–9 am, 10–11 am, 12–1 pm, 2–3 pm, and 4–5 pm). During each observation period, the presence of each behavior (at least once) in 3 min segments was scored by a well-trained observer (20 segments/period × 6 periods = 120 segments per dam per day). Out of 120 segments per day, we calculated the percentage of segments in which the presence of each behavior was scored. As the maternal behavior to be analyzed, we focused on LG, because LG has shown to be a maternal behavioral trait that is matrilineally transmitted to the next generation in rats (Francis et al., 1999; Champagne et al., 2003). In rat pups, contact with mother stimulates hypothalamic oxytocin neurons that are important for the development of social behavior (Kojima et al., 2012). Therefore, we analyzed LG, contact with pups, and leaving pups in the present study.

### 2.6 Fecal sample collection and microbiota solution preparation

Fresh fecal samples from randomly selected donor mice (a few grains of feces per mice) were directly collected in sterilized collection tubes on PD56, suspended in phosphate-buffered saline containing 20% glycerol, immediately frozen using liquid nitrogen, and stored at −80°C until further use. The fecal sample (a grain of feces) was thawed on ice immediately before administration and filtered using a mesh 100 μm cell strainer followed by centrifugation. The supernatant was removed, and the remaining pellets were dissolved in 400 μL of sterilized saline. The solution was transferred to a sterilized vinyl isolator for subsequent oral administration. The solution (100 μL) was forcibly administered to each mouse by oral sonde.

### 2.7 Statistical analyses

Results are expressed as mean ± SEM. All statistical analyses were performed using the JMP (Ver.13, SAS Institute Inc., NC, USA). In experiments 1 and 3, two-way repeated-measures analysis of variance (ANOVA) was performed with group and postpartum days as factors. In Experiment 2, a three-way repeated-measures ANOVA was performed with prenatal mother, postnatal mother, and postpartum day as factors. For the LG in Experiments 2 and 3 and the leaving pups in Experiment 3, in which the distribution of data was better fitted by a lognormal distribution than by a normal distribution, log-transformed data were used in the analysis.

## 3 Results

In this study, we observed maternal behaviors and analyzed the incidence of each behavior in female mice from 1 to 9 days postpartum. In all the experiments, the incidence of LG and contact with pups decreased gradually from 1 to 9 days postpartum. In contrast, the incidence of leaving pups increased gradually from 1 to 9 days postpartum. Repeated-measures ANOVA showed a main effect of postpartum day for all observation items in all mouse groups in all the experiments (Figures 1–3).

### 3.1 Experiment 1: observations of maternal behavioral traits across two generations

In F1 female mice, two-way repeated-measures ANOVA showed that the main effect of the group was observed in the LG, and there was no interaction between group and postpartum day in the LG (Figure 1B). As shown in Figure 1B, LG expression was lower in EW-F1 mice than in NW-F1 mice during the observation period. However, the main effect of group was not observed in case of either contact with pups or leaving pups (Figures 1C,D). There was no significant interaction between group and postpartum day in either contact with pups or leaving pups.

In F2 female mice, two-way repeated-measures ANOVA showed that the main effect of group was observed in the LG, and there was no significant interaction between group and postpartum day in the LG (Figure 1E). Similar to that in F1 female mice, LG expression was lower in EW-F2 mice than in NW-F2 mice during the observation period. There were no main effects of group or significant interaction between group and postpartum day with respect to contact with pups and leaving pups (Figures 1F,G).

### 3.2 Experiment 2: cross-fostering experiments on F2 female offspring

Two-way repeated-measures ANOVA showed that the main effect and interactions on the observed behaviors of F1 female mice in experiment 2 were similar to those of F1 female mice in experiment 1 (Figures 2A–C). The main effect of group was only observed in the LG, and there was no interaction between group and postpartum day in the LG. As shown in Figure 2A, LG expression was lower in EW-F1 mice than in NW-F1 mice during the observation period. The three-way repeated ANOVA that analyzed the cross-foster experiment of F2 females showed that, regarding LG, the main effect was seen in postnatal mothers but not in prenatal mothers (Figure 2E). There were no interactions in any combination of factors, including the interaction between the postnatal mother and prenatal mother. As shown in Figure 2E, LG expression was lower in NW-EW and EW-EW mice than in NW-NW and EW-NW mice during the observation period. In other words, LG expression in F2 female mice, the postnatal mothers of which were EW-F1 mice, was lower than that in mice, for which the postnatal mothers were NW-F1 mice, regardless of whether the prenatal mother was EW-F1 or NW-F1. However, there were no main effects in either the prenatal mother or postnatal mother with respect to contact with pups and leaving pups (Figures 2F,G). In these behaviors, there were no interactions in any combination of factors, except for the interaction between prenatal mother and postpartum day.

### 3.3 Experiment 3: transplantation of fecal microbiota into GF mice

The two-way repeated-measures ANOVA showed that the main effect of the group was observed in the LG, and there was no interaction between group and postpartum day in the LG (Figure 3B). As shown in Figure 3B, LG expression was lower in GF-EW mice than in GF-NW mice during the observation period. However, the main effect of the group was not observed for either contact with pups or leaving pups (Figures 3C,D). There was no interaction between the group and postpartum day in either contact with pups or leaving pups.

## 4 Discussion

In both Experiments 1 and 2, the ANOVA for EW-F1 and NW-F1 mothers indicated that the LG expression of both mothers decreased with postpartum days, but LG expression in EW-F1 mice was consistently lower than that in NW-F1 mice from postnatal days 1 to 9. These results suggested that, similar to the findings of our previous studies (Kikusui et al., 2005; Sakamoto et al., 2021), mouse mothers experiencing early weaning had lower LG expression than mothers who experienced normal weaning. In Experiment 1, ANOVA for F2 mice also indicated that the LG expression of EW-F2 mice was lower than that of NW-F2 mice, although both F2 mice were weaned normally, similar to the observation for F1 mice. This suggests that the matrilineal transmission of maternal behavioral traits occurred for LG expression in mice. Furthermore, in the cross-fostering experiment in Experiment 2, ANOVA showed that there was a main effect in postnatal mothers but not in prenatal mothers for LG expression. In addition, there was no interaction between postnatal mother and prenatal mother. Considering these, together with the fact that LG expression was lower in NW-EW and EW-EW mice than in NW-NW and EW-NW mice, it is indicated that LG expression was lower in mice raised by postnatal mothers who experienced early weaning, regardless of the weaning mode of the prenatal mother. This suggests that the intergenerational transmission of maternal LG traits observed in mice is dependent on the postnatal environment and that the traits are similar to those of a nurturing mother.

Our findings in mice are similar to those obtained in rats. LG expression levels in rats were highly correlated with the LG expression levels in their mother (Francis et al., 1999; Champagne et al., 2003). Cross-fostering experiments have suggested that LG expression levels are more similar in nurturing mothers than in biological mothers (Francis et al., 1999; Champagne et al., 2003). Similarly with these findings, it is suggested that the matrilineal transmission of maternal behavioral traits occurred for only LG but not for contact with pus or leaving pups in the present study. Among maternal behavioral traits, LG behavioral traits may be commonly passed on to the next generation of rodents. The reasons for only LG behavioral traits being transmitted to the next generation remain unclear. However, while the frequency and duration of contact with pups is difficult to change due to the need to maintain the pup’s body temperature and breastfeed, it is thought that LG is relatively easy to change. In addition, LG stimulation received from the mother during the developmental period has a strong impact on offspring development may be other possible reason. For example, rodent offspring raised under low-LG stimulation show the following phenotypes. In rats, offspring of low-LG mothers have endocrine hypersensitivity to stress due to reduced glucocorticoid receptors in the hippocampus, which are important for negative feedback regulation of the stress endocrine response (Francis et al., 1999). Behaviorally, rat offspring of low-LG mothers have been shown to exhibit high anxiety (Caldji et al., 1998). In mice, we have previously revealed that the offspring of EW mothers are hypersensitive to pain (Sakamoto et al., 2021) and skin development is also affected, with high resistance to skin barrier disruption (Sakamoto et al., 2019). As these phenotypes are necessary for survival in harsh environments, the intergenerational transmission of maternal LG traits may be one of the strategies by which rodent species adapt to their environment. The intergenerational transmission of maternal behavioral traits, which is also observed in humans and primates (Fairbanks, 1989; Benoit and Parker, 1994; Miller et al., 1997; Maestripieri and Carroll, 1998; Maestripieri, 2005), may be a common phenomenon in mammals. Further studies on the implications of the transgenerational effects of maternal care are required.

As an important postnatal environment for the intergenerational transmission of maternal LG traits, this study focused on the maternal microbiota and hypothesized that the vertical transmission of gut microbiota is involved in this process. In experiment 3, maternal behavioral traits were analyzed in GF mice colonized with gut microbiota from NW-F1 and EW-F1 mice, and the ANOVA showed that LG expression in GF-EW mice was consistently lower than that in GF-NW mice from postnatal days 1 to 9, similar to the difference in LG expression between NW-F2 and EW-F2 mice. Furthermore, no effects were found on maternal behavioral traits other than LG, as in NW-F2 and EW-F2. Considering that the colonization of gut microbiota of NW-F1 and EW-F1 mice with GF mother mice allows their pups to mimic the differences in maternal LG traits of NW-F2 and EW-F2 mice, it is strongly suggested that vertical transmission of gut microbiota is involved in the transmission of maternal behavioral traits, although we did not analyze the gut microbiota profiles. Although the early acquisition of microbiota in offspring may be influenced by pregnancy, delivery, and after birth (Mady et al., 2023), the results of our GF mouse and cross-fostering studies suggest that the postnatal acquisition of microbiota is mainly involved in the intergenerational transmission of this maternal behavioral trait. Although the route of transmission of the maternal gut microbiota to the pups remains unclear, the possible route may be transmission upon the pups licking the feces of the mother mouse.

Studies on effects of microbiota on social behavior in mice have mainly focused on the effects on sociability and social novelty (Desbonnet et al., 2014; Buffington et al., 2016; Wu et al., 2021; Di Gesù et al., 2022). As for the effects on maternal behavior in mice, a particular intestinal *E. coli* strain has been reported to suppress maternal behavior, including LG, and increase non-maternal behavior in studies examining the pathological effects of *E. coli* caused by malnutrition and inhibition of weight gain in offspring (Lee et al., 2021). To the best of our knowledge, this study is the first to suggest the involvement of microbiota in individual-level differences in maternal behavioral traits. Future studies should focus on how colonization of the microbiota in childhood regulates LG expression in adulthood. In early studies using GF mice, an enhanced hypothalamic–pituitary–adrenal (HPA) stress response seen in GF mice was shown to be corrected by reconstitution of SPF microbiota only in the early postnatal stage, suggesting a window in which the microbiota can modulate the development of HPA-axis (Sudo et al., 2004). Similarly, previous studies on mice have suggested the existence of a window in which the microbiota can modulate social development (Buffington et al., 2016; Di Gesù et al., 2022). In this study, colonization of the microbiota in GF pups only occurred after the pups were 10 days old; however, there were differences in maternal LG expression between GF-NW and GF-EW mice, such as NW-F2 and EW-F2. This suggests that the window in which the microbiota can modulate maternal LG behavior remains open at 10 days old. This window would be examined by varying the timing of microbiota colonization in GF pups. For the differences in brain development related to maternal LG expression, the role of methylation levels of the ERα promoter in MPOA has been shown in rats (Champagne et al., 2006). The mechanism by which the gut microbiota epigenetically alters brain development, including whether the same phenomenon occurs in mice as in rats, needs to be investigated in the future.

In summary, our results clearly suggest that, among maternal behavioral traits, LG is intergenerationally transmitted in mice and that vertical transmission of the gut microbiota is involved in this process. This study demonstrates the universality of the intergenerational transmission of maternal behavioral traits and sheds new light on the interplay between maternal behavior and the gut microbiota. In the future, it will be important to investigate what differences in the gut microbiota between NW and EW mice affect the expression of LG.

## Data availability statement

The original contributions presented in the study are included in the article/Supplementary material, further inquiries can be directed to the corresponding authors.

## Ethics statement

The animal study was approved by Animal Experiment Committee of Azabu University. The study was conducted in accordance with the local legislation and institutional requirements.

## Author contributions

KM: Conceptualization, Data curation, Funding acquisition, Writing – original draft. UA: Investigation, Writing – review & editing. NF: Investigation, Writing – review & editing. KT: Investigation, Writing – review & editing. MK: Investigation, Writing – review & editing. MM: Investigation, Writing – review & editing. MY: Investigation, Writing – review & editing. IK: Investigation, Writing – review & editing. SK-A: Data curation, Writing – original draft. TK: Conceptualization, Data curation, Funding acquisition, Writing – original draft.
